# Socio-ecological correlates of children’s diet in the first 2 years of life: influence of socio-economic position and migration background in a French birth cohort

**DOI:** 10.1017/S136898002610281X

**Published:** 2026-06-03

**Authors:** Marion Lecorguillé, Alexandra Descarpentrie, Laura Pavicic, Camille Le Gal, Stéphanie Vandentorren, Priscille Sauvegrain, Barbara Heude, Marie-Aline Charles, Blandine de Lauzon-Guillain, Sandrine Lioret

**Affiliations:** 1 Université Paris Cité and Université Sorbonne Paris Nord, Inserm, INRAE, Center for Research in Epidemiology and StatisticS (CRESS), Paris, France; 2 Department of Pediatrics, The Saban Research Institute, University of Southern California, Los Angeles, CA, USA; 3 Santé Publique France, French National Public Health Agency, Saint-Maurice, France; 4 Centre INSERM U1219 Bordeaux Population Health Research Center, University of Bordeaux, Bordeaux, France; 5 Department of Maïeutics, Sorbonne Université, Paris, France; 6 Ined, Inserm, EFS, joint unit ELFE, Aubervilliers, France

**Keywords:** child diet, parental social determinants, socio-economic position, immigrants

## Abstract

**Objective:**

Strong social inequalities exist in chronic diseases related to diet and lifestyle. However, few studies have comprehensively examined the social determinants of children’s diet at the family level, including paternal characteristics and migration background. This study aimed to assess the associations between family-based socio-ecological factors and children’s dietary patterns (DP) during the first 2 years of life.

**Design:**

This longitudinal observational study applied a socio-ecological model to explore associations with children’s DP. We conducted multivariable linear regression analyses with DP – identified via principal component analysis – as outcomes, sequentially adjusting for maternal factors: immigrant status, education, older children in the household, single motherhood and age (block 1); occupation (block 2) and household income (block 3). Similar analyses were performed with paternal characteristics.

**Setting:**

Nationwide French ELFE birth cohort.

**Participants:**

We used data from an FFQ completed by parents of 12 696 2-year-old children.

**Results:**

Children born to mothers with a low socio-economic position (low education level; manual worker, unemployed or student or in the lowest household income quintile), with previous children, or who were living as a single parent had low scores on a DP defined as ‘*Balanced and diversified*’. These children also showed greater adherence to a ‘*Discretionary consumption*’ pattern, as did those born to immigrant mothers. Overall, consistent findings were observed with paternal socio-economic factors.

**Conclusions:**

Early-life dietary inequalities are shaped by family-level social determinants, emphasising the need for early nutritional interventions tailored to disadvantaged families, particularly those with a migration background, low socio-economic status or single parenthood.

## Introduction

Strong health social inequalities still exist in high-income countries for chronic diseases (e.g. obesity) that are partly explained by lifestyle factors, including dietary intake^(1,2)^. Over the past 3 or 4 decades, the nutrition transition in high-income countries has been accompanied by a marked Westernisation of diets, characterised by an increased consumption of energy-dense, nutrient-poor foods^(3,4)^. This shift has occurred along with an increase in sedentary behaviours and a decline in levels of physical activity^(5)^. Furthermore, studies have shown that dietary patterns (DP) are socio-economically differentiated from the first year of life^(6–9)^ and tend to track over the life course^(10–12)^, thus contributing to the early setting of socio-economic inequalities in overweight^(13,14)^.

So far, most studies evaluating the social determinants of children’s diet have focused on maternal education, household income and, to a lesser extent, maternal occupational status as indicators of the family’s socio-economic position (SEP)^(9,15–17)^. Some studies suggest that maternal education has a stronger influence on child health and nutrition than paternal education^(18)^. However, only a few have considered paternal factors to measure SEP, which represents a gap in the literature given the increasing involvement of fathers in child care^(9)^.

Besides maternal and paternal SEP, additional factors contributing to the family social vulnerability have been understudied and deserve consideration to address inequalities more comprehensively^(7,14,19)^. In particular, immigrants’ health status tends to decline once they are settled in the host country^(20,21)^. On the one hand, immigrants initially experience the ‘healthy migrant effect’: those who are able to migrate tend to have better health status and SEP on average than their non-migrating counterparts^(21)^. On the other hand, primarily because of unfavourable working and living conditions in the host country, stereotypes and discrimination, social downgrading and difficulties in accessing health care, immigrants may face various barriers to maintaining their health advantage^(20,21)^. Furthermore, immigrants may undergo an acculturation process by adopting certain aspects of the lifestyle of the host country (e.g. Western diet), which often differ from their original socio-cultural background and traditions^(22)^. However, how these paradigms and challenges affect their children’s diet is unclear. Furthermore, studies that have examined the social determinants linked to both parents, while properly addressing their complementarities, are scarce. To address this analytic challenge, socio-ecological models have been developed as a framework to better account for the complexity and intricate nature of contextual and individual characteristics associated with a given outcome, for example, children’s diet^(23)^.

In this context, we used a family socio-ecological framework to investigate the associations of various social determinants with DP of children in the first 2 years of life, while also accounting for immigrant status, in the French nationwide ELFE (*Étude Longitudinale Française depuis l’Enfance*) birth cohort. We conducted two separate analyses to disentangle the independent associations of maternal and paternal socio-ecological factors on the child’s diet.

## Methods

This study is reported according to the Strengthening the Reporting of Observational Studies in Nutritional Epidemiology (STROBE-nut) guidelines (see Supplementary STROBE-nut statement).

### Study population

This research project was based on the ELFE study, a French nationwide birth cohort with more than 18 000 children included at birth, the project’s rationale and design were previously detailed^(24)^. Participants were recruited over 25 days, divided into four selected periods (waves) spanning all four seasons in 2011. The inclusion criteria were birth at ≥33 weeks’ amenorrhoea, singleton or twin birth and mother >18 years old, who gave informed consent and did not plan to leave mainland France within 3 years. To ensure the inclusion of women with limited French literacy, the information letter and consent form were translated into Arabic, Turkish and English, the three most commonly spoken languages of mothers giving birth in France. Participation in the cohort was proposed to women who gave birth in 349 maternity hospitals randomly selected among the 544 public and private maternity hospitals in mainland France. Among eligible mothers, 51 % agreed to participate (*N* 18 040).

### Data collection and measurement

Data for this study were obtained from several questionnaires administered at inclusion in the study and follow-ups. The main parental socio-economic, socio-cultural and socio-demographic characteristics were collected from a telephone interview with parents 2 months after their child(ren)’s birth. Other data were collected via face-to-face interviews conducted by trained investigators at the maternity hospital and from medical records at birth. To assess children’s dietary intake, we used data collected in a food introduction questionnaire at age 9 months and a food frequency questionnaire (FFQ) at age 2 years. The questionnaires were self-completed by parents on paper or online (at age 9 months) and via phone call (at age 2 years). The present manuscript focuses on DP at age 2 years; however, analyses conducted at age 9 months are described in the Supplementary file (Supplementary Text 1 and Supplementary Tables 2–3 and 5–6).

#### Child dietary patterns at age 2 years

A twenty-item FFQ was used at age 2 years, with six possible responses ranging from ‘never’ to ‘several times a day’ (‘never’ was coded zero; ‘less often’ as 1 time/month; ‘several times a month’ as three times/months; ‘several times a week’ as twelve times/month; ‘once a day’ as thirty times/month and ‘several times a day’ as sixty/month). This FFQ was broadly consistent with the questionnaire previously used in the French EDEN cohort^(9,11)^ and was designed to capture the child’s overall diet in terms of major food and beverage groups. Based on similarities in food type, energy density and context of consumption, food and beverage items were classified into sixteen groups (Supplementary Table 1). Frequencies within each group were summed and standardised (Supplementary Table 1). We included children with at least one available food item, which resulted in a sample of *N* 12 907 children at age 2 years.

#### Family-based socio-ecological factors

The mother’s immigrant status was classified in three groups: ‘non-immigrant’ for women born French to two French parents (whether in or outside France); ‘descendant of immigrants’ for women born in France to at least one non-French parent and ‘immigrant’ for women born outside France without French nationality at birth. The three indicators of the mother’s SEP were defined as follows:

- Education level: low (≤ high school level), intermediate (1–2 years university degree) and high (≥ 3-year university degree).
- Occupational category: manual worker, unemployed or student; clerk; farmer or skilled blue-collar worker; intermediate occupation (e.g. teacher, nurse, technician and foreman) and executive or top management.
- Household income per consumption unit: calculated according to the definition from the French National Institute of Statistics and Economic Studies, with distinct coefficients applied to each household member (1 consumption unit for the householder; 0.5 for other members aged ≥ 14 years and 0.3 for each child <14 years). The income was then categorised into quintiles.

Other socio-demographic variables included maternal age at delivery (in years); single motherhood (yes or no, defined by whether the mother was living with the father/partner); number of older siblings of the ELFE child in the household (including half-siblings: no siblings; 1 sibling or ≥ 2 siblings). Conurbation size was classified in three groups (rural <2000 habitants; urban 2000 to < 200 000 habitants or ≥ 200 000 habitants). Similar variables were established for fathers, including immigrant status, education, age and occupation, classified as above.

### Statistical analyses

#### Dietary patterns derivation

DP analysis has become widespread in characterising the overall diet of individuals^(25)^. Instead of analysing individual foods or nutrients, this approach is useful to account for combinations and interactions of various foods containing multiple nutrients. We included the sixteen standardised dietary variables and used principal component analysis (PCA) to derive the child DP. Analyses were conducted on the maximum sample, including twins, with missing dietary intake data imputed using the regularized iterative PCA method from the R package missMDA^(26)^. The number of relevant principal components (DPs) was selected considering eigenvalues >1·0, the scree plot and interpretability^(25)^. The PCA factor loadings with absolute value >0·30 were considered to contribute meaningfully to the principal component. To enhance the interpretation of the components, the PCA was performed with varimax rotation (orthogonal transformation)^(27)^. Individual scores for each DP were calculated by summing the observed standardised values for each dietary variable weighted according to the PCA factor loadings. In a sensitivity analysis, we also conducted PCA for the subsample of children with < 25 % missing data on the FFQ (*N* 12 901 at age 2 years), which gave consistent findings.

#### Hierarchical regression models

**Figure 1.**
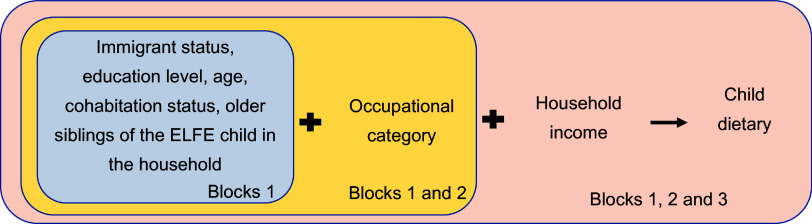
Hierarchical linear regression models, conceptual framework of family-based socio-ecological correlates associated with child dietary patterns. Two separate analyses are conducted: one focusing on maternal factors and the other on paternal factors. The three embedded models were adjusted for child sex, conurbation size and the partner’s education level, which was included as a confounder.

The socio-ecological factors under study were structured within a three-nested block framework as follows: immigrant status, education level, age, single motherhood, older siblings of the ELFE child (**block 1**); occupational category (**block 2**) and household income (**block 3**). We hypothesised that education level would be an upstream factor, influencing occupational category, which in turn would affect income^(14)^. These social determinants were identified according to both factors highlighted in the literature as influencing a child’s diet and their availability in the cohort^(9,11,28)^. Hierarchical linear regression analyses were then conducted to examine the associations between maternal socio-ecological factors (independent variables) and child DP (outcomes)^(29)^. Following the socio-ecological approach, variables were added in blocks, from the most distal to the most proximal, and interpreted in the first model in which they were included (Fig. 1)^(14,29)^. This method ensures that intermediate/downstream variables (potential mediators) do not affect the associations between distal/upstream factors and the outcomes. We created these three embedded models separately for maternal factors and paternal factors. All models were adjusted for child sex and conurbation size as covariates and for partner’s education level as a confounder. Regression analyses were performed after randomly excluding one twin from each pair in the database (*N* 12 696 at age 2 years). Missing data for socio-ecological factors were imputed by using the R ‘MICE’ package. We generated twenty imputed datasets, using logistic regression, the multinomial logit model and predictive mean matching for categorical and quantitative variables, respectively^(30)^ (see Supplementary Table 7). Analyses of complete cases (*N* 10 836 at age 2 years) gave similar results (Supplementary Table 8). We further tested an interaction term between immigrant status and education level, with the assumption that the influence of SEP on child DP might differ depending on the parents’ immigrant status (p_interaction_ < 0·05 considered statistically significant).

## Results

### Participant characteristics

Characteristics of the ELFE study participants at baseline are in Table 1. Mothers and fathers were about 31 and 33 years old on average. Among mothers, 10·2 % were immigrants and 10·5 % descendants of immigrants; these rates were 5·4 % and 16·9 % for fathers. Parents born outside France mainly were from North Africa, Turkey and Sub-Saharan Africa. About one-third of parents had a high education level, and about one-fifth had executive or top management jobs. The socio-demographic characteristics of the sample at child age 2 years were consistent with those at baseline. However, the proportion of parents with high SEP (whether measured by education, occupation or income) was higher at the 2-year follow-up than at baseline, as was the proportion of non-immigrants.

**Table 1.** Characteristics of the ELFE cohort population at baseline (*N* 17 933) and at the 2-year follow-up (*N* 12 696)^*^

	ELFE population				Sample at 2 years	
	Missing data		Population		Population	
	%	*n*	% or mean	*n* or sd	% or mean	*n* or sd
***Maternal characteristics***						
**Age at delivery (years)**	0·5	87	30·6	5·1	31·18	4·7
**Primiparous**	1·5	264	45·9	8113	45·8	5733
Number of older siblings of the ELFE child in the household^**^	9·2	1657				
None			44·5	7243	44·0	5482
1 sibling			36·5	5939	37·5	4678
≥ 2 siblings			19·0	3094	18·5	2309
**Single motherhood: no**	10·0	1792	95·7	15 446	97·2	12 034
**Immigrant status**						
Immigrant	10·0	1791	10·2	1651	9·2	1143
Descendant of immigrants			10·5	1690	10·1	1254
Non-immigrant			79·3	12 801	80·7	10 040
Country of birth^***^	0·2	39				
France			87	15 568	90	11 422
European Union (excluding France)			2·2	390	2·1	271
North Africa and Turkey			4·9	879	3·2	411
Sub-Saharan Africa			3·7	659	2·8	356
Other			2·2	398	1·9	236
**Education level**	10·7	1914				
Low (≤ high school)			41·8	6700	35·3	4349
Intermediate (1- to 2-year university)			22·1	3534	23·7	2920
High (≥ 3-year university)			36·1	5785	41·1	5067
**Employment at child age 2 months**	10·0	1794				
Employed/self-employed			71·4	11 524	76·0	9466
**Occupational category**	4·1	741				
Manual worker, unemployed, student			6·6	1134	4·8	608
Clerk			49·0	8431	45·5	5736
Farmer and skilled blue-collar worker			3·8	661	3·7	468
Middle occupation			23·0	3953	25·7	3237
Executive and top management			17·5	3013	20·2	2547
**Household income per unit consumption (Euros/month)**	14·7	2641				
First quintile (lowest)			20·1	3071	15·7	1897
Second quintile			19·8	3030	19·1	2299
Third quintile			17·0	2601	17·8	2144
Fourth quintile			22·9	3496	24·8	2983
Fifth quartile (highest)			20·2	3094	22·6	2728
***Paternal characteristics***						
**Age at delivery (years)**	4·8	861	33·3	6·2	33·7	5·9
**Immigrant status**						
Immigrant	25·8	4621	5·4	722	3·6	403
Descendant of immigrants			16·9	2253	15·9	1771
Non-immigrant			77·7	10 337	80·4	8943
Country of birth^***^	4·1	737				
France			87·8	15 100	89·6	11 216
European Union (excluding France)			1·8	318	1·9	240
North Africa and Turkey			5·7	980	4·4	549
Sub-Saharan Africa			3·1	540	2·7	341
Other			1·5	258	1·4	174
**Education level**	24·1	4323				
Low (≤ high school)			49·3	6703	45·8	5205
Intermediate (1- to 2-year university)			18·0	2450	19	2161
High (≥ 3-year university)			32·7	4457	35·3	4011
**Employment at child age 2 months**	2·4	424				
Employed/self-employed			89·1	15 598	91·4	11 453
**Occupational category**	4·7	844				
Manual worker, unemployed, student			15·7	2678	12·9	1608
Clerk			38·1	6517	36·3	4513
Farmer and skilled blue-collar worker			10·9	1858	10·4	1295
Middle occupation			13·3	2265	14·9	1853
Executive and top management			22·1	3771	25·5	3172
**Conurbation size**	10·1	1811				
Rural			21·6	3480	22·9	2846
Urban < 200 000 inhabitants			31·6	5102	31·4	3898
≥ 200 000 inhabitants			46·8	7540	45·7	5682
**Child sex**	0·1	10				
Boy			51·4	9219	50·8	6446
Girl			48·6	8704	49·2	6250

* Selection on having at least one information available for the socio-ecological correlate variables.

** Number of older siblings of the ELFE child (including half-siblings).

*** Country of birth: France; European Union (except France); Turkey and Maghreb (Morocco, Algeria and Tunisia); Sub-Saharan Africa and Other (other Africa, Eastern/Central Europe, Asia, South/Central America grouped together because of small number of observations. SD standard deviation.

### Dietary patterns resulting from principal component analysis

For children aged 2 years, the first two DP explained 28 % of the total variance (Table 2). The first pattern, ‘*Diversified and balanced*’, was characterised by a high intake of starchy foods, bread, meat and eggs, fish, cheese, cooked and raw vegetables, fresh fruits and compotes. The second pattern, ‘*Discretionary consumption*’ had high positive factor loadings for discretionary sweet and savory foods, French fries, processed meat and sugar-sweetened beverages and negative factor loadings for cooked vegetables.

**Table 2.** Principal component analysis (PCA) factor loadings for the dietary patterns at age 2 years^*^ (*N* 12 907)

	Dietary patterns		Consumption frequency (/day)	
	Diversified and balanced	Discretionary consumption	Median	IQR
***Dietary variables***				
Starchy foods	0·61^**^	−0·09	1·0	0·43–1
Breakfast cereals	0·02	0·14	0·10	0–1·0
Bread	0·34^**^	0·21	1·0	0·43–2
Meat and eggs	0·60^**^	0·19	1·1	0·53–1·43
Fish	0·33^**^	0·15	0·43	0·10–0·43
Cheese	0·35^**^	0·14	1·0	0·43–2·0
Dairy products: yogurts and « petits suisses »	0·29	0·03	2·0	1·0–2·0
Raw vegetables	0·39^**^	0·15	0·10	0·03–0·43
Cooked vegetables, including soup	0·70^**^	−0·31^**^	1·0	0·43–2·0
Fresh fruits	0·54^**^	−0·01	0·43	0·43–1·0
Compotes	0·37^**^	−0·02	1·0	0·43–1·0
Discretionary sweet foods	0·09	0·59^**^	1·03	0·53–1·43
Discretionary savory foods	0·05	0·68^**^	0·13	0·07–0·20
French fries	0·05	0·68^**^	0·10	0·03–0·10
Processed meat	0·09	0·54^**^	0·03	0–0·10
Sugar-sweetened beverages	0·03	0·62^**^	0·43	0·07–1·0
Explained variation, %	0·14	0·14		

ELFE birth cohort study. IQR, interquartile range.

* PCA with varimax rotation.

** Absolute value of factor loadings ≥ 0.30.

### Associations of family socio-ecological factors with children’s dietary patterns

At age 2 years, children born to immigrant mothers, with a low SEP (i.e. low and, to a lesser extent, intermediate education level; manual worker, unemployed or student, and to a lesser extent, clerk; first quintile of household income) or experiencing single motherhood were less likely to follow a ‘*Diversified and balanced*’ DP, as did those with older siblings (Table 3). Children born to mothers with a low SEP (as above) scored high on the ‘*Discretionary consumption*’ DP, as were those born to younger or immigrant mothers (and to a lesser extent those born to descendants of immigrants) or with older siblings. Effect sizes were overall larger with this second DP.

**Table 3.**
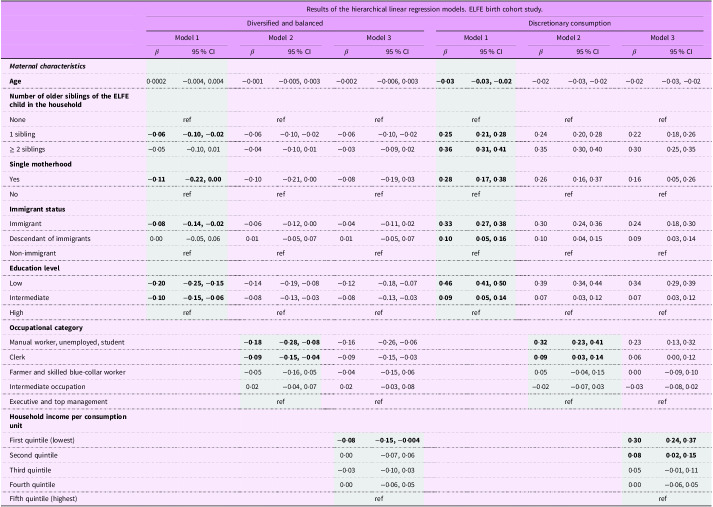
Association of maternal socio-ecological factors with dietary patterns in children aged 2 years (*N* 12 696)

Model 1 included maternal age, number of older siblings of the ELFE child in the household, single motherhood, immigrant status and education level; model 2 additionally included occupational category and model 3 additionally included household income. All models were adjusted for child sex and conurbation size as covariates and partner’s education level as a confounder. Coefficients are interpreted when the variable appears the first time (in grey), but we showed all estimates for indication.

For the association of paternal socio-ecological factors with the two DP, we found smaller effect sizes for SEP indicators (education and occupation) than those for mothers (Table 4). Paternal immigrant status was not associated with the ‘*Diversified and balanced*’ DP, unlike maternal immigrant status. However, as compared with mothers, father’s immigrant status showed a stronger gradient with the ‘*Discretionary consumption*’ DP.

**Table 4.**
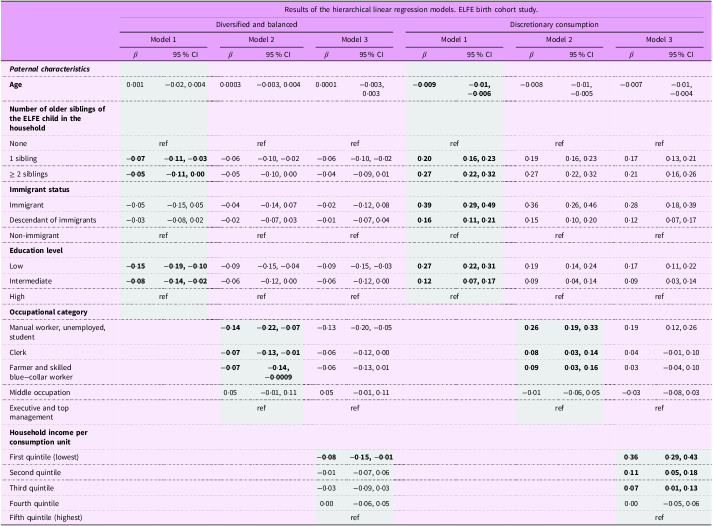
Association of paternal socio-ecological factors with dietary patterns in children aged 2 years (*N* 12 696) Table 4 long description.

Model 1 included paternal age, number of older siblings of the ELFE child in the household, immigrant status and education level; model 2 additionally included occupational category and model 3 additionally included household income. All models were adjusted for child sex and conurbation size as covariates and partner’s education level and single motherhood as confounders. Coefficients are interpreted when the variable appears the first time (in grey), but we showed all estimates for indication.

Additionally, we found a significant interaction between maternal immigrant status and education level on the child’s ‘*Discretionary consumption*’ pattern (p_interaction_ = 0·001), so we stratified our analyses accordingly (Table 5). The inverse associations of maternal education level and household income with ‘*Discretionary consumption*’ DP scores were stronger for children born to descendants of immigrants and immigrants *v*. non-immigrants. Conversely, the association between maternal occupational category and the ‘*Discretionary consumption*’ DP was specific to non-immigrant mothers (i.e. greater adherence in children born to manual workers, unemployed mothers or students).

**Table 5.** Maternal socio-ecological correlates of the ‘discretionary consumption’ dietary pattern at age 2 years, stratified by migration status

	Immigrant status					
	Non-immigrant (N 8932)		Descendant of migrants (N 1060)		Immigrant (N 844)	
	*β*	95 % CI	*β*	95 % CI	*β*	95 % CI
**Age**	−**0·02**	−**0.03,** −**0.02**	−**0·03**	−**0.05,** −**0.02**	−**0·03**	−**0.05,** −**0.01**
**Number of older siblings of the ELFE child in the household**						
None	ref		ref		ref	
1 sibling	**0·24**	**0·20, 0·28**	**0·22**	**0·08, 0·37**	**0·28**	**0·10, 0·47**
≥ 2 siblings	**0·32**	**0·27, 0·38**	**0·35**	**0·17, 0·54**	**0·61**	**0·39, 0·83**
**Single motherhood**						
Yes	0·21	−0.07, 0.49	0·46	−0.19, 1.11	0·07	−0.59, 0.72
No	ref		ref		ref	
**Education level**						
Low	**0·42**	**0·37, 0·46**	**0·61**	**0·44, 0·77**	**0·53**	**0·33, 0·72**
Intermediate	**0·08**	**0·03, 0·12**	**0·11**	−**0.06, 0.28**	**0·23**	**0, 0·46**
High	ref		ref		ref	
**Occupational category**						
Manual worker, unemployed, student	**0·32**	**0·21, 0·43**	−0·19	−0.59, 0.21	0·24	−0.1, 0.57
Clerk	**0·11**	**0·05, 0·16**	0·05	−0.15, 0.26	−0·05	−0.29, 0.19
Farmer and skilled blue-collar worker	0·08	−0.03, 0.18	0·01	−0.35, 0.37	0·01	−0.43, 0.45
Middle occupation	0·003	−0.05, 0.06	−0·13	−0.33, 0.07	−0·18	−0.44, 0.07
Executive and top management	ref		ref		ref	
**Household income**						
First quintile (lowest)	**0·26**	**0·18, 0·34**	**0·38**	**0·13, 0·62**	**0·39**	**0·13, 0·66**
Second quintile	**0·08**	**0·02, 0·15**	−0·03	−0.27, 0.21	0·15	−0.12, 0.42
Third quintile	0·05	−0.01, 0.11	0·01	−0.21, 0.22	−0·07	−0.36, 0.22
Fourth quintile	−0·01	−0.07, 0.04	−0·03	−0.22, 0.17	−0·03	−0.29, 0.24
Fifth quintile (highest)	ref		ref		ref	

Results are presented following the hierarchical regression approach (on complete cases) with coefficients interpreted when the variable appears the first time. Model 1 included maternal age, number of older siblings of the ELFE child in the household, single motherhood and education level; model 2 additionally included maternal occupational category and model 3 additionally included household income (all three models were additionally adjusted for paternal education level, child sex and conurbation size as covariates).

## Discussion

### Summary of results

Using longitudinal data, we provide novel and comprehensive insights into the social determinants of children’s diets during the first 2 years of life. We examined a range of SEP indicators for both mothers and fathers and also considered parental migration background, living context and household composition. At age 2 years, children with older siblings, and those born to immigrant mothers, with a low SEP, or who were raising their child alone, had lower scores on the ‘*Diversified and balanced*’ DP. Conversely, these children had higher scores on the ‘*Discretionary consumption*’ pattern, as did children born to immigrant mothers (and, to a lesser extent, descendants of immigrants). We observed consistent associations for socio-ecological factors related to fathers; however, effect sizes were smaller for SEP, and the gradient associated with immigrant status was steeper for the ‘*Discretionary consumption*’ DP. Importantly, our findings indicate that social inequalities in children’s diets are visible as early as 9 months of age, particularly for the ‘Discretionary consumption’ DP (Supplementary Tables 5 and 6), highlighting the perinatal period as a critical window of opportunity for equity-based interventions.

### Interpretation

Consistent with previous studies^(9,16,17)^, we confirmed the presence of early socio-economic inequalities in the child’s diet quality across various indicators, including the education and occupation of both parents, as well as household income. However, the socio-ecological approach allowed us to further account for their interrelation, along with broader family characteristics likely to influence the child’s diet. Our findings, adjusted for the partner’s education level, support the notion that maternal and paternal education are not merely proxies for each other but have independent and possibly synergistic effects on a child’s diet. Parents with high educational attainment are more likely to understand and apply nutritional information (food literacy), thereby leading to better feeding practices, healthier eating behaviours and role modelling during family meals^(31,32)^. However, we found that the influence of paternal education level, and more broadly the father’s SEP, on a toddler’s diet was weaker than that of the mother. The beta values for maternal low and intermediate education level in terms of the ‘*Diversified and Balanced*’ pattern at age 2 years were 25 % and 20 % higher, respectively, than those for fathers. Previous research has consistently shown that maternal education is more strongly associated with children’s dietary diversity, and with antenatal and post-natal care, than paternal education^(18)^. One possible explanation is that, regardless of their partner’s education level, fathers may be less engaged in daily childcare routines, including early feeding practices, whereas maternal education may influence a broader range of nutrition-related behaviours^(18)^.

Along with education, white-collar occupations, often providing social networks and support, and higher income, facilitating access to healthier, yet more expensive foods, serve as complementary and essential levers in empowering parents to adopt healthy diets^(1)^. Education, employment and income could also indicate residence in a more resource-rich neighbourhood, characterised by better proximity, density and diversity of food retailers. In the present study, the associations of education and occupation with the ‘*Diversified and balanced*’ pattern remained significant, albeit attenuated, after adjustment for downstream SEP indicators. This finding highlights their importance, along with knowledge, food literacy and social interactions, in promoting a healthy diet, beyond the influence of income.

Of note, the association between income and DP did not follow a graded trend, as observed with education, but instead showed a threshold effect. A minimum food budget is required to achieve a nutritionally adequate diet^(33,34)^; therefore, this finding suggests that below a certain income level, budgetary trade-offs come into play, compromising diet quality. Families facing financial constraints are more likely to experience food insecurity, which often leads to the purchase of energy-dense, nutrient-poor foods, as these are generally more affordable^(31,35)^.

Beyond SEP indicators, few studies have reported on the effect of immigrant status on a child’s early diet. Consistent with our findings, the IDEFICS study, conducted across eight European countries, showed that children with a migrant background (defined by the authors as having at least one parent born outside the cohort country) were more likely to be assigned to the processed foods cluster DP at baseline (2–9 years old) and follow-up (4–11 years old)^(7)^. However, previous findings from the ELFE cohort revealed that immigrant mothers, as well as descendants of immigrants, had healthier, less processed diets during their pregnancy than non-immigrant mothers^(20)^. This observation suggests dietary discrepancies within households with a migration history. Ethno-cultural identity, the desire to preserve culinary traditions and beliefs and representations are psychosocial factors that can foster greater adherence to a traditional and healthy diet among immigrants^(22)^. However, migration is often accompanied by conflicting social norms, worsened socio-economic conditions, challenges related to language and health literacy and limited access to affordable, culturally appropriate foods. Although these challenges can be mitigated by community networks in the host country, they still create significant barriers to maintaining a traditional diet for all family members in the host country^(36)^. Most research on nutrition issues in children from immigrant families has been conducted in the USA, with limited insights available from other countries^(37,38)^. Qualitative studies highlight that although immigrant mothers may undergo dietary Westernisation at a relatively slow pace, their children often develop a preference for unhealthy processed foods, more quickly; this shift can lead to the rejection of traditional foods from their parents’ country of origin^(39)^. In our data, this hypothesis would apply to the older siblings of the ELFE child (if any), who are more exposed to external influences, including junk food advertisements^(40)^, and are therefore more likely to challenge the parent’s adherence to dietary guidelines^(9)^. In fact, we found the presence of older siblings in the household inversely associated with the quality of the ELFE child’s DP. This association remained when income was taken into account, so it is not merely a question of budgetary trade-offs linked to a larger family. Of note, unlike mothers, immigrant fathers in the ELFE study did not adhere to healthier diets as compared with non-immigrant fathers^(41)^. This observation may suggest that immigrant fathers are less committed to preserving a traditional and healthy diet and may be more permissive of their children’s Westernised food choices. Our findings support this hypothesis, as reflected in the steeper gradient of paternal immigrant status associated with the ‘*Discretionary consumption*’ DP as compared with maternal immigrant status.

Additionally, the persistence of an association between immigrant status and the ‘*Discretionary consumption*’ DP, even after adjusting for the three dimensions of SEP, highlights a direct effect of migration that is independent of SEP. This observation confirms that consuming discretionary foods is not solely driven by educational attainment, type of occupation and income but also involves complex socio-cultural processes. Whether these processes are influenced by factors such as country of birth, reasons for migration, migration trajectory, duration of residence in France, the built environment or psychosocial aspects, including experiences of discrimination, anxiety, stress and limited access to culturally appropriate food and healthcare in the host country, warrants further investigation and is beyond the scope of the present analysis^(42,43)^. Still, the analysis stratified by immigrant status revealed steeper inverse gradients for the ‘*Discretionary consumption*’ pattern with maternal education and income among children born to mothers with a migrant *v*. non-migrant background. This finding suggests that the intersection of economic and ethno-cultural vulnerabilities linked to migration may synergistically shape the child’s diet quality, a phenomenon some authors have termed the ‘double penalty’^(44)^.

Finally, the association between occupational category and the ‘*Discretionary consumption*’ pattern was more pronounced among non-immigrant than immigrant mothers. This finding likely reflects the occupational downgrading among immigrant women, defined in the context of international migration as the ‘loss in occupational status between one’s last job in the home country and first job in the host country’^(45)^. Consequently, occupational classification may more accurately capture a mother’s actual professional status in the non-immigrant population, whereas the relative ‘misclassification’ among women with a migratory history could obscure a strong association between these variables.

Previous studies have also found young maternal age and the presence of siblings at home negatively related to diet quality in children ≤ 7 years old^(9,16,17)^. Generational differences in lifestyle and a potentially lower prioritisation of healthy eating behaviours among relatively younger adults have been suggested as possible explanations^(46,47)^. Additionally, having multiple children often imposes greater time constraints on parents, thus reducing the time available for purchasing and preparing healthy meals^(9)^. This challenge is even more pronounced in single-parent families, as our results confirm.

### Public health implications

Given the early establishment of DP and their tracking across the life course^(11)^, implementing early and effective strategies to improve dietary intake is crucial for preventing childhood obesity and, in turn, reducing the risk of related non-communicable diseases in adulthood. Our findings highlight the importance of involving both parents in family-based nutritional interventions, as well as other members who may serve as role models, such as older siblings^(48,49)^. They also underscore the need to consider the broader social context of the families, not only to identify the most vulnerable groups that should be reached in priority but also to tailor and personalise interventions according to the specific barriers these families face in adopting or maintaining healthy eating habits^(37)^.

Universal obesity prevention and health promotion programmes have largely been ineffective when relying primarily on information provision because they fail to address the diverse and specific needs of the most vulnerable populations, therefore exacerbating the existing social inequalities in health^(49,50)^. There is growing advocacy for co-designing interventions with stakeholders, including target populations, in that participatory approaches are essential for gaining a deeper understanding of family realities, challenges, expectations and representations. Such co-creative methods also help foster a trusting relationship between delivery agents and end users^(49,51)^. Some studies have additionally shown that interventions targeting allophone minorities are more effective when they are delivered by bilingual agents (or supported by interpreters), when they respect cultural beliefs and practices, and when they are developed in partnership with the community or lay agents^(49,52)^. Effectiveness of interventions also seemed enhanced with the involvement of multidisciplinary teams, such as social workers and healthcare professionals^(49,51)^. Last, but not least, addressing structural determinants with policies that enhance geographical access to healthy foods and services, such as regulating the density of fast-food restaurants relative to fresh produce markets, while also improving employment conditions, purchasing power, and the affordability of nutritious foods, is crucial^(49)^. These structural measures are essential for empowering individuals to adopt and sustain healthier behaviours.

### Limitations and strengths

Although social desirability bias and dietary assessment errors cannot be entirely ruled out, the identification of two well-established DP, diversified (more balanced at age 2 years) and discretionary, at both 9 months (Supplementary Table 3) and 2 years of age, along with consistent socio-ecological associations, reinforces the robustness and validity of our results. The originality of this study is its holistic approach to the social determinants of early-life diets, even though it does not encompass all levels of the socio-ecological model, including broader environmental factors such as area deprivation or the social and community context, and downstream factors, such as time constraints, household equipment, mobility and other financial burdens, particularly housing costs. Additionally, socio-ecological factors were primarily collected at age 2 months: although we cannot rule out that some factors, such as occupation and household income, may have changed by age 2 years, we chose to maintain consistency by using the same variables at both times (i.e. 9 months and 2 years). While our socio-ecological framework assumes a specific ordering between education and income, we acknowledge that these factors are embedded in broader intergenerational processes of social inequality and could be framed within alternative causal pathways; however, when both factors were included in the same model (model 3), the associations were only slightly attenuated and remained significant, supporting the robustness of our findings. Finally, as is often the case in population-based cohorts, there is a potential selection bias at baseline and differential attrition during subsequent follow-ups in the ELFE cohort. Consequently, families with a migrant background and those in more socio-economically disadvantaged situations are likely under-represented. Nevertheless, the study still captures a range of socio-economic and socio-cultural variability, which suggests that the effect sizes would probably have been even greater if the most vulnerable population sub-groups had been better represented.

## Conclusion

These findings provide novel and comprehensive insights into the social determinants contributing to the development of early-life dietary inequalities, particularly those operating at the family level (i.e. mother, father and siblings). To be more effective, nutritional interventions should be designed to target and adapt to the needs of the most disadvantaged families, especially those with a migration background or a low SEP or experiencing single parenthood. To mitigate the cumulative effects of social inequalities over the life course, such interventions should start as early as pregnancy and early childhood.

## Supporting information

10.1017/S136898002610281X.sm001 Lecorguillé et al. supplementary material Lecorguillé et al. supplementary material

## Data Availability

Some or all datasets generated during and/or analysed during the current study are not publicly available but are available from the corresponding author on reasonable request and signing of the appropriate data-sharing agreement and approval by the steering committee of the cohort.

## Supplementary material

For supplementary material accompanying this paper visit https://doi.org/10.1017/S136898002610281X

## Table 4. Long description

The table presents the results of hierarchical linear regression models for the ELFE birth cohort study, examining the association of paternal socio-ecological factors with two dietary patterns: Diversified and balanced, and Discretionary consumption. The table has 26 rows and 12 columns. Column headers include Model 1, Model 2, and Model 3 for both Diversified and balanced and Discretionary consumption, with each model showing beta (β) and 95% confidence interval (CI). Row labels include Paternal characteristics such as Age, Number of older siblings of the ELFE child in the household, Immigrant status, Education level, Occupational category, and Household income per consumption unit. Each row provides beta values and 95% confidence intervals for the three models under each dietary pattern. Notable trends include the association of paternal immigrant status with the Discretionary consumption dietary pattern, showing a stronger gradient compared to the Diversified and balanced pattern. Education level and occupational category also show varying effects on the dietary patterns.

Navigate back to Table 4..
