# Supplementary material for: Socio-ecological correlates of children’s diet in the first 2 years of life: influence of socio-economic position and migration background in a French birth cohort
Source: Public Health Nutr. 2026 Jun 3;29(1):e136. doi: 10.1017/S136898002610281X (PMC13434481; doi:10.1017/S136898002610281X)
Supplement: Supplementary file 1 [file S136898002610281Xsup001.docx]

**Supplementary Text 1: Child dietary patterns (DPs) at 9 months and their associations with family socio-ecological factors**

*Child DPs*

We used data for children aged 9 months from a food introduction questionnaire, comprising 29 items with five possible responses ranging from “not yet introduced” to “every day or almost every day” (“not yet introduced” was coded zero; “once” as 1 time/month; “several times” as 4 times/month; “often” as 13 times/month; and “every day or almost every day” as 30 times/month). According to similarities in food type, energy density and context of consumption, food and beverage items were classified into 12 groups (**Supplementary Table 2**), with the frequencies of foods within each group summed. We included the 12 standardized dietary variables for 9,070 children, each with at least one available dietary item, in a principal component analyses (PCA) to derive the children’s DPs (1). Missing data for dietary intake were imputed with the regularized iterative PCA method and the R missMDA package (2). The number of relevant principal components (DPs) was selected considering eigenvalues > 1.0, the scree plot, and interpretability (1). PCA factor loadings with an absolute value > 0.30 were considered to have a reasonable contribution to the principal component. Individual scores for each DP were calculated by summing the observed standardized values for each dietary variable weighted according to the PCA factor loadings.

For children aged 9 months, the three identified DPs accounted for 48% of the total variance. The first DP, labelled **“***Diversified*”**,** was characterized by high consumption frequency across all groups, except for sugar-sweetened beverages and infant cereals and was slightly less balanced compared to the one observed at 2 years (**Supplementary Table 3**). It was only slightly negatively correlated with age of complementary feeding introduction and positively with the “*Diversified/Balanced*” DP identified at 2 years (**Supplementary Table 4**; Pearson correlation <0.20, p-value <0.001). The second DP showed high factor loadings for bread, cheese, discretionary sweet foods, sugar-sweetened beverages but negative factor loadings for vegetables, fruits, and fish: it was labelled “*Discretionary consumption*” (**Supplementary Table 3**). It was positively correlated with the similar DP identified at age 2 years (Pearson correlation=0.23, p-value <0.001) (**Supplementary Table 4**). We also identified a third pattern with positive factor loadings for water, infant cereals, and yogurts and negative factor loadings for bread: it was labelled “*Infant cereals and yogurt”* **Supplementary Table 3**) and was negatively correlated with any breastfeeding duration (Pearson correlation=-0.18, p<0.001) (**Supplementary Table 4**). Consistent with a previous finding showing that boys are more likely to be introduced to complementary feeding earlier than girls (3), we observed that boys scored higher on the “*Diversified*” DP than girls. This result led us to consistently adjust for child sex in multivariable analyses.

*Associations of family socio-ecological factors with children’s DPs*

We performed hierarchical regression analyses using multiple imputation (N=8,961 at 9 months). The family-based socio-ecological correlates of the “*Discretionary consumption*” DP were consistent at both 9 months and 2 years. We reported consistent associations with the second DP that aligned with the “*Discretionary consumption*” pattern found at 2 years (**Supplementary Tables 5 and 6**).Children born to parents with a low socio-economic position (SEP), immigrants, and to a lesser extent, descendants of immigrants, were more likely to follow this DP. Associations differed partly with the “*Diversified*” DP (**Supplementary Table 5 and 6**), likely because, at 9 months, this pattern was less healthy (balanced) than at 2 years and represented an overall earlier introduction to foods. Specifically, we observed higher scores on this pattern only among 9-month-old children born to younger parents, mothers of immigrant descent, or mothers with an intermediate education level. No other associations were observed with this pattern, except a negative link with the presence of older siblings at home. Lastly, parents with low SEP (low education level; first quintile of household income), farmers, skilled blue-collar workers, single parents, or those with previous children were less likely to provide a “*Infant cereals and yogurt*” diet to their child at age 9 months, as did immigrant parents (and to a lesser extent, descendants of immigrants). Analyses of complete cases (N=7,889 at 9 months) showed similar results (not shown, available on request).

**Supplementary Table 1**: **Food groups considered for the principal component analysis (PCA) at 2 years of age.**

| **Food groups** | **Food items (where relevant*)** |
| --- | --- |
| Sugar-sweetened beverages | - Fruit juices - Sweetened drinks (syrup, soda) |
| Yogurts or « petits suisses » | NA |
| Infant/toddler cereals, breakfast cereals | NA |
| Bread | NA |
| Starchy food | NA |
| Meat and eggs | - Meat, ham - Eggs |
| Fish | NA |
| Cheese | NA |
| Cooked vegetables (without potatoes), including soup | NA |
| Raw vegetables | NA |
| Fresh fruits | NA |
| Compotes | NA |
| Discretionary sweet foods | - Viennese pastries, cookies, cakes - Other sweets |
| French fries | NA |
| Processed meat (except ham) | NA |
| Discretionary savory foods | - Quiches, pizzas, savory pies - Chips |

***Food items are specified only when several items were grouped to define a food or beverage group.**

**NA: not applicable**

**Supplementary Table 2**: **Food groups considered for the principal component analysis (PCA) at 9 months of age.**

| **Food groups** | **Food items (where relevant*)** |
| --- | --- |
| Sugar-sweetened beverages | - Fruit juices - Sweetened drinks (syrup, soda) |
| Water | Bottle and tap water |
| Yogurts or « petits suisses » | NA |
| Infant cereals | NA |
| Bread | NA |
| Starchy food | - Potatoes - Pasta, rice |
| Meat and eggs | - Meat, ham - Egg white or yolk |
| Fish | NA |
| Cheese | NA |
| Vegetables | - Green beans - Carrots, pumpkin - Peas - Artichokes, spinach - Other vegetables |
| Fruits | - Apples, pears - Bananas - Strawberries, raspberries - Peaches, apricots - Other fruits |
| Discretionary sweet foods | - Cookies - Other sweet desserts |

***Food items are specified only when several items were grouped to define a food or beverage group.**

**NA: not applicable**

**Supplementary Table 3: Principal component analysis (PCA) factor loadings for the dietary patterns derived at 9 months (N=9070).**

|  |  | **Dietary patterns** |  | |
| --- | --- | --- | --- | --- |
|  | **Diversified** | **Discretionary consumption** | **Infant cereals and yogurt** | |
| ***Dietary variables*** |  |  |  |  |
| Water | 0.38* | 0.24 | 0.44* |  |
| Starchy foods | 0.69* | -0.19 | -0.12 |  |
| Infant cereals | 0.22 | 0.24 | 0.65* |  |
| Bread | 0.40* | 0.44* | -0.31* |  |
| Meat and eggs | 0.69* | -0.16 | 0.03 |  |
| Fish | 0.65* | -0.32* | 0 |  |
| Cheese | 0.42* | 0.37* | -0.22 |  |
| Yogurts and « petits suisses » | 0.48* | 0.10 | 0.46* |  |
| Vegetables | 0.70* | -0.35* | -0.16 |  |
| Fruits | 0.66* | -0.29* | -0.01 |  |
| Discretionary sweet foods | 0.50* | 0.48* | -0.19 |  |
| Sugar-sweetened beverages | 0.25 | 0.48* | -0.17 |  |
| **Explained variation, %** | 0.28 | 0.11 | 0.09 |  |

ELFE birth cohort study

^a^ PCA with varimax rotation; * Absolute value of factor loadings ≥ 0.30

**Supplementary Table 4: Pearson correlations and P values between the identified dietary patterns at different ages, breastfeeding duration and age at complementary feeding variables (N= 7902)**

|  | DPs at 2 years | |
| --- | --- | --- |
|  | *“Diversified and balanced*” | “*Discretionary consumption*” |
| DPs at 9 months |  |  |
| *“Diversified*” | 0.15** | 0.09** |
| “*Discretionary consumption*” | -0.08** | 0.23** |
| “*Infant cereals and yogurt*” | -0.02* | -0.09** |
|  |  |  |
|  | Breastfeeding duration | Age at complementary feeding |
| Breastfeeding duration |  | 0.23** |
| DPs at 9 months |  |  |
| *“Diversified*” | -0.08** | -0.19** |
| “*Discretionary consumption*” | 0.01 | -0.18** |
| “*Infant cereals and yogurt*” | -0.18** | -0.04** |
| DPs at 2 years |  |  |
| *“Diversified and balanced*” | 0.07** | 0.06** |
| “*Discretionary consumption*” | -0.06** | -0.18** |

Recommendations for interpreting these correlation coefficients are as follows: low, <0.3; moderate, 0.3–0.6; and high, >0.6. *P < 0.05, **P < 0.001

Breastfeeding duration (any breastfeeding) in months.

Age at complementary feeding in months.

**Supplementary Table 5: Maternal socioecological correlates of the child’s dietary patterns at age 9 months (N=8,961).**

|  | **Results of the hierarchical linear regression models, β (95% CI). ELFE birth cohort study** | | | | | | | | |
| --- | --- | --- | --- | --- | --- | --- | --- | --- | --- |
|  | **Diversified** | | | **Discretionary consumption** | | | **Infant cereals and yogurt** | | |
|  | **Model 1** | **Model 2** | **Model 3** | **Model 1** | **Model 2** | **Model 3** | **Model 1** | **Model 2** | **Model 3** |
| **Age** | **-0.04**  **(-0.05, -0.03)** | -0.04  (-0.05, -0.03) | -0.04  (-0.05, -0.03) | **-0.01**  **(-0.02, -0.01)** | -0.01  (-0.02, -0.01) | -0.01  (-0.02, -0.01) | -0.002  (-0.008, 0.003) | -0.003  (-0.008, 0.003) | -0.003  (-0.008, 0.003) |
| **Number of older siblings of the ELFE child in the household** |  |  |  |  |  |  |  |  |  |
| None | ref | ref | ref | ref | ref | ref | ref | ref | ref |
| 1 sibling | **-0.21**  **(-0.30, -0.12)** | -0.21  (-0.30, -0.12) | -0.20  (-0.29, -0.11) | **0.12**  **(0.06, 0.17)** | 0.11  (0.06, 0.17) | 0.10  (0.04, 0.15) | **-0.17**  **(-0.22, -0.12)** | -0.17  (-0.22, -0.12) | -0.17  (-0.22, -0.12) |
| ≥ 2 siblings | **-0.27**  **(-0.39, -0.16)** | -0.27  (-0.39, -0.15) | -0.24  (-0.36, -0.11) | **0.27**  **(0.20, 0.34)** | 0.26  (0.19, 0.34) | 0.22  (0.15, 0.30) | **-0.27**  **(-0.34, -0.20)** | -0.27  (-0.33, -0.20) | -0.25  (-0.32, -0.18) |
| **Single motherhood** |  |  |  |  |  |  |  |  |  |
| Yes | -0.07  (-0.37, 0.22) | -0.06  (-0.35, 0.24) | 0.00  (-0.30, 0.30) | **0.42**  **(0.25, 0.59)** | 0.41  (0.23, 0.58) | 0.33  (0.16, 0.51) | **-0.20**  **(-0.36, -0.04)** | -0.19  (-0.35, -0.03) | -0.15  (-0.31, 0.01) |
| No | ref | ref | ref | ref | ref | ref | ref | ref | ref |
| **Immigrant status** |  |  |  |  |  |  |  |  |  |
| Immigrant | -0.01  (-0.16, 0.14) | 0.00  (-0.15, 0.16) | 0.03  (-0.13, 0.18) | **0.23**  **(0.13, 0.32)** | 0.21  (0.12, 0.30) | 0.17  (0.08, 0.27) | **-0.30**  **(-0.38, -0.21)** | -0.29  (-0.37, -0.20) | -0.27  (-0.36, -0.18) |
| Descendant of immigrants | **0.18**  **(0.05, 0.32)** | 0.18  (0.05, 0.32) | 0.19  (0.05, 0.32) | 0.07  (-0.01, 0.15) | 0.07  (-0.02, 0.15) | 0.06  (-0.02, 0.14) | **-0.08**  **(-0.16, -0.003)** | -0.08  (-0.15, -0.001) | -0.07  (-0.15, 0.003) |
| Non-immigrant | ref | ref | ref | ref | ref | ref | ref | ref | ref |
| **Education level** |  |  |  |  |  |  |  |  |  |
| Low | 0.07  (-0.04, 0.17) | 0.07  (-0.05, 0.19) | 0.09  (-0.03, 0.21) | **0.21**  **(0.15, 0.28)** | 0.16  (0.09, 0.23) | 0.13  (0.05, 0.20) | **-0.09**  **(-0.15, -0.03)** | -0.08  (-0.15, -0.02) | -0.07  (-0.13, 0.0004) |
| Intermediate | **0.10**  **(0.00, 0.20)** | 0.09  (-0.01, 0.20) | 0.09  (-0.01, 0.20) | 0.05  (-0.02, 0.11) | 0.02  (-0.04, 0.09) | 0.02  (-0.05, 0.08) | -0.01  (-0.07, 0.04) | -0.01  (-0.07, 0.05) | -0.01  (-0.07, 0.05) |
| High | ref | ref | ref | ref | ref | ref | ref | ref | ref |
| **Occupational category** |  |  |  |  |  |  |  |  |  |
| Manual worker, unemployed, student |  | -0.18  (-0.42, 0.05) | -0.14  (-0.38, 0.10) |  | **0.22**  **(0.08, 0.36)** | 0.14  (-0.01, 0.28) |  | -0.12  (-0.25, 0.01) | -0.10  (-0.24, 0.03) |
| Clerk |  | 0.03  (-0.10, 0.15) | 0.04  (-0.09, 0.17) |  | **0.09**  **(0.02, 0.17)** | 0.06  (-0.02, 0.14) |  | 0.00  (-0.07, 0.07) | -0.01  (-0.08, 0.06) |
| Farmer and skilled blue-collar worker |  | 0.01  (-0.22, 0.23) | 0.03  (-0.20, 0.26) |  | 0.07  (-0.07, 0.21) | 0.01  (-0.13, 0.15) |  | **-0.24**  **(-0.37, -0.11)** | -0.23  (-0.36, -0.10) |
| Middle occupation |  | 0.02  (-0.10, 0.13) | 0.02  (-0.10, 0.14) |  | 0.03  (-0.04, 0.10) | 0.01  (-0.06, 0.08) |  | -0.03  (-0.10, 0.03) | -0.05  (-0.11, 0.02) |
| Executive and top management |  | ref | ref |  | ref | ref |  | ref | ref |
| **Household income per consumption unit** |  |  |  |  |  |  |  |  |  |
| 1st quintile (lowest) |  |  | -0.15  (-0.31, 0.02) |  |  | **0.27**  **(0.17, 0.37)** |  |  | -0.07  (-0.17, 0.02) |
| 2nd quintile |  |  | -0.06  (-0.20, 0.08) |  |  | **0.11**  **(0.03, 0.20)** |  |  | 0.02  (-0.06, 0.10) |
| 3rd quintile |  |  | 0.00  (-0.13, 0.14) |  |  | 0.07  (-0.01, 0.15) |  |  | 0.05  (-0.03, 0.12) |
| 4th quintile |  |  | 0.01  (-0.10, 0.13) |  |  | 0.06  (-0.01, 0.13) |  |  | 0.05  (-0.02, 0.12) |
| 5th quintile (highest) |  |  | ref |  |  | ref |  |  | ref |

Model 1 included maternal age, number of older siblings of the ELFE child in the household, single motherhood, immigrant status, education level; model 2 additionally included occupational category; and model 3 additionally included household income. All models were adjusted for child sex and conurbation size as covariates and partner’s education level as a confounder. Coefficients are interpreted when the variable appears the first time (in grey) but we showed all estimates for indication.

**Supplementary Table 6: Paternal socio-ecological correlates of the child’s dietary patterns at age 9 months (N=8,961).**

|  | **Results of the hierarchical linear regression models, β (95% CI). ELFE birth cohort study** | | | | | | | | |
| --- | --- | --- | --- | --- | --- | --- | --- | --- | --- |
|  | **Diversified** | | | **Discretionary consumption** | | | **Infant cereals and yogurt** | | |
|  | **Model 1** | **Model 2** | **Model 3** | **Model 1** | **Model 2** | **Model 3** | **Model 1** | **Model 2** | **Model 3** |
|  |  |  |  |  |  |  |  |  |  |
| **Age** | **-0.02**  **(-0.02, -0.01)** | -0.02  (-0.02, -0.01) | -0.02  (-0.02, -0.01) | -0.002  (-0.006, 0.003) | -0.0006  (-0.005, 0.004) | 0.0009  (-0.004, 0.005) | -0.004  (-0.008, 0.000) | -0.004  (-0.008, 0.0002) | -0.004  (-0.008, 0.0002) |
| **Number of older siblings of the ELFE child in the household** |  |  |  |  |  |  |  |  |  |
| None | ref | ref | ref | ref | ref | ref | **ref** | ref | ref |
| 1 sibling | **-0.26**  **(-0.35, -0.17)** | -0.26  (-0.35, -0.17) | -0.26  (-0.34, -0.17) | **0.08**  **(0.03, 0.13)** | 0.08  (0.03, 0.13) | 0.06  (0.01, 0.11) | **-0.17**  **(-0.21, -0.12)** | -0.17  (-0.21, -0.12) | -0.16  (-0.21, -0.11) |
| ≥ 2 siblings | **-0.37**  **(-0.49, -0.26)** | -0.37  (-0.49, -0.26) | -0.35  (-0.47, -0.24) | **0.20**  **(0.13, 0.27)** | 0.20  (0.13, 0.27) | 0.15  (0.08, 0.23) | **-0.26**  **(-0.32, -0.19)** | -0.26  (-0.32, -0.19) | -0.24  (-0.31, -0.18) |
| **Immigrant status** |  |  |  |  |  |  |  |  |  |
| Immigrant | -0.01  (-0.27, 0.25) | -0.01  (-0.27, 0.25) | 0.02  (-0.25, 0.28) | **0.21**  **(0.05, 0.37)** | 0.20  (0.04, 0.36) | 0.13  (-0.03, 0.29) | **-0.25**  **(-0.39, -0.10)** | -0.24  (-0.39, -0.09) | -0.21  (-0.36, -0.06) |
| Descendant of immigrants | 0.00  (-0.12, 0.12) | 0.00  (-0.12, 0.12) | 0.01  (-0.11, 0.13) | **0.10**  **(0.02, 0.17)** | 0.09  (0.02, 0.16) | 0.07  (0.00, 0.14) | **-0.15**  **(-0.21, -0.08)** | -0.15  (-0.21, -0.08) | -0.14  (-0.20, -0.07) |
| Non-immigrant | ref | ref | ref | ref | ref | ref | ref | ref | ref |
| **Education level** |  |  |  |  |  |  |  |  |  |
| Low | 0.03  (-0.07, 0.13) | 0.03  (-0.09, 0.15) | 0.03  (-0.09, 0.15) | **0.18**  **(0.12, 0.24)** | 0.12  (0.05, 0.20) | 0.10  (0.03, 0.18) | 0.02  (-0.04, 0.07) | 0.04  (-0.03, 0.10) | 0.04  (-0.03, 0.10) |
| Intermediate | 0.03  (-0.08, 0.14) | 0.03  (-0.09, 0.15) | 0.03  (-0.09, 0.15) | **0.12**  **(0.05, 0.19)** | 0.10  (0.02, 0.17) | 0.09  (0.01, 0.16) | **0.08**  **(0.02, 0.14)** | 0.09  (0.02, 0.15) | 0.08  (0.02, 0.15) |
| High | ref | ref | ref | **ref** | ref | ref | ref | ref | ref |
| **Occupational category** |  |  |  |  |  |  |  |  |  |
| Manual worker, unemployed, student |  | 0.02  (-0.15, 0.18) | 0.03  (-0.14, 0.19) |  | **0.19**  **(0.09, 0.29)** | 0.13  (0.02, 0.23) |  | -0.06  (-0.15, 0.03) | -0.05  (-0.14, 0.04) |
| Clerk |  | 0.00  (-0.12, 0.12) | 0.00  (-0.13, 0.12) |  | **0.08**  **(0.00, 0.15)** | 0.03  (-0.04, 0.11) |  | -0.01  (-0.08, 0.06) | -0.01  (-0.08, 0.06) |
| Farmer and skilled blue-collar worker |  | -0.03  (-0.19, 0.12) | -0.02  (-0.18, 0.14) |  | **0.10**  **(0.00, 0.19)** | 0.03  (-0.06, 0.13) |  | **-0.09**  **(-0.17, 0.00)** | -0.08  (-0.16, 0.01) |
| Middle occupation |  | -0.04  (-0.17, 0.08) | -0.05  (-0.18, 0.08) |  | 0.02  (-0.06, 0.09) | -0.01  (-0.09, 0.07) |  | 0.01  (-0.07, 0.08) | 0.00  (-0.07, 0.07) |
| Executive and top management |  | ref | ref |  | ref | ref |  | ref | ref |
| **Household income per consumption unit** |  |  |  |  |  |  |  |  |  |
| 1st quintile (lowest) |  |  | -0.09  (-0.26, 0.07) |  |  | **0.31**  **(0.21, 0.41)** |  |  | **-0.10**  **(-0.19, -0.01)** |
| 2nd quintile |  |  | -0.02  (-0.16, 0.13) |  |  | **0.14**  **(0.06, 0.23)** |  |  | 0.01  (-0.07, 0.09) |
| 3rd quintile |  |  | 0.04  (-0.09, 0.18) |  |  | **0.09**  **(0.01, 0.17)** |  |  | 0.04  (-0.04, 0.12) |
| 4th quintile |  |  | 0.04  (-0.07, 0.16) |  |  | **0.08**  **(0.01, 0.15)** |  |  | 0.05  (-0.02, 0.11) |
| 5th quintile (highest) |  |  | ref |  |  | ref |  |  | ref |

Model 1 included paternal age, number of older siblings of the ELFE child in the household, immigrant status, education level; model 2 additionally included paternal occupational category; and model 3 additionally included household income. All models were adjusted for child sex, conurbation size as covariates and partner’s education level and single motherhood as confounders. Coefficients are interpreted when the variable appears the first time (in grey) but we showed all estimates for indication.

**Supplementary Table 7: List of variables included for imputation and proportion of missing data in ELFE***

| **Variable** | **Type of variable** | **Model used to predict missing data** | **Missing data %** |
| --- | --- | --- | --- |
| Maternal age | Continuous | Predictive mean matching | 0.5 |
| Paternal age | Continuous | Predictive mean matching | 4.8 |
| Maternal immigrant status | Categorical (3 categories) | Multinomial regression | 10 |
| Maternal country of birth | Categorical (5 categories) | Multinomial regression | 0.2 |
| Paternal immigrant status | Categorical (3 categories) | Multinomial regression | 25.8 |
| Paternal country of birth | Categorical (5 categories) | Multinomial regression | 4.2 |
| Maternal education | Categorical (3 categories) | Ordinal regression | 10.7 |
| Paternal education | Categorical (3 categories) | Ordinal regression | 24.1 |
| Maternal employment | Binary | Logistic regression | 10 |
| Paternal employment | Binary | Logistic regression | 2.4 |
| Maternal occupational category | Categorical (5 categories) | Multinomial regression | 4.1 |
| Paternal occupational category | Categorical (5 categories) | Multinomial regression | 4.7 |
| Household income | Categorical (5 categories) | Ordinal regression | 14.7 |
| Single motherhood | Binary | Logistic regression | 10 |
| Number of older siblings of the ELFE child in the household | Categorical (3 categories) | Ordinal regression | 9.2 |
| Parity | Binary | Logistic regression | 1.5 |
| Social welfare health insurance | Binary | Logistic regression | 0.9 |
| Professional medical follow-up during pregnancy | Categorical (3 categories) | Multinomial regression | 1.2 |
| Maternal pre-pregnancy BMI | Continuous | Predictive mean matching | 1.8 |
| Paternal BMI at inclusion | Continuous | Predictive mean matching | 31 |
| Gestational weight gain | Continuous | Predictive mean matching | 2.5 |
| Maternal smoking pregnancy | Categorical (3 categories) | Ordinal regression | 1.6 |
| Paternal smoking pregnancy | Categorical (3 categories) | Ordinal regression | 30.3 |
| Gestational weight gain | Continuous | Predictive mean matching | 4.8 |
| Maternal pregnancy DASH | Continuous | Predictive mean matching | 15.1 |
| Conurbation size | Categorical (3 categories) | Ordinal regression | 10.1 |
| Child sex | Binary | Logistic regression | 0.06 |
| Cohort period of recruitment | Categorical (5 categories) | Multinomial regression | 0 |

*This table lists all the variables used for the imputation step and the prediction of missing data on our variables of interest. (We used several variables such as lifestyle and pregnancy follow-up to predict missing data on the variables used in our final model).

**Supplementary Table 8: Parental socio-ecological correlates of the child dietary patterns at age 2 years (complete cases N=10,836 for maternal model and N=10,206 for paternal model).**

|  | **Results of the hierarchical linear regression models, β (95% CI). ELFE birth cohort study** | | | | | |
| --- | --- | --- | --- | --- | --- | --- |
|  | **Diversified and balanced** | | | **Discretionary consumption** | | |
|  | **Model 1** | **Model 2** | **Model 3** | **Model 1** | **Model 2** | **Model 3** |
| **Maternal characteristics** |  |  |  |  |  |  |
| **Age** | 0.0005  (-0.004, 0.005) | -0.001  (-0.006, 0.003) | -0.002  (-0.007, 0.003) | **-0.02 (-0.03, -0.02)** | -0.02 (-0.03, -0.02) | -0.02  (-0.023, -0.015) |
| **Number of older siblings of the ELFE child in the household** |  |  |  |  |  |  |
| None | ref | ref | ref | ref | ref | ref |
| 1 sibling | **-0.08 (-0.12, -0.03)** | -0.07 (-0.12, -0.03) | -0.07 (-0.11, -0.02) | **0.25 (0.21, 0.28)** | 0.24 (0.20, 0.28) | 0.22 (0.18, 0.26) |
| ≥ 2 siblings | -0.05 (-0.11, 0.01) | -0.04 (-0.10, 0.02) | -0.03 (-0.09, 0.03) | **0.36 (0.31, 0.41)** | 0.35 (0.30, 0.40) | 0.30 (0.25, 0.35) |
| **Single motherhood** |  |  |  |  |  |  |
| Yes | -0.06 (-0.32, 0.19) | -0.06 (-0.31, 0.20) | -0.01 (-0.27, 0.24) | 0.22 (-0.01, 0.45) | 0.21 (-0.02, 0.44) | 0.09 (-0.14, 0.32) |
| No | ref | ref | ref | ref | ref | ref |
| **Immigrant status** |  |  |  |  |  |  |
| Immigrant | **-0.08 (-0.15, -0.01)** | -0.06 (-0.13, 0.02) | -0.04 (-0.11, 0.03) | **0.26 (0.20, 0.32)** | 0.24 (0.17, 0.30) | 0.19 (0.12, 0.25) |
| Descendant of immigrants | -0.01 (-0.07, 0.05) | -0.01 (-0.07, 0.06) | 0.00 (-0.07, 0.06) | **0.11 (0.05, 0.17)** | 0.11 (0.05, 0.16) | 0.10 (0.04, 0.15) |
| Non-immigrant | ref | ref | ref | ref | ref | ref |
| **Education level** |  |  |  |  |  |  |
| Low | **-0.20 (-0.25, -0.15)** | -0.13 (-0.19, -0.08) | -0.12 (-0.18, -0.06) | **0.45 (0.41, 0.50)** | 0.39 (0.33, 0.44) | 0.34 (0.28, 0.39) |
| Medium | **-0.11 (-0.16, -0.06)** | -0.09 (-0.14, -0.04) | -0.09 (-0.14, -0.03) | **0.09 (0.05, 0.14)** | 0.07 (0.02, 0.12) | 0.07 (0.02, 0.12) |
| High | ref | ref | ref | ref | ref | ref |
| **Occupational category** |  |  |  |  |  |  |
| Manual worker, unemployed, student |  | **-0.29 (-0.40, -0.17)** | -0.25 (-0.37, -0.14) |  | **0.27 (0.17, 0.37)** | 0.18 (0.07, 0.28) |
| Clerck |  | **-0.09 (-0.15, -0.03)** | -0.08 (-0.15, -0.02) |  | **0.09 (0.04, 0.15)** | 0.07 (0.01, 0.12) |
| Farmer and skilled blue-collar worker |  | -0.05 (-0.16, 0.06) | -0.03 (-0.14, 0.08) |  | 0.08 (-0.02, 0.17) | 0.02 (-0.08, 0.12) |
| Intermediate occupation |  | 0.02 (-0.04, 0.08) | 0.03 (-0.03, 0.09) |  | -0.02 (-0.07, 0.04) | -0.03 (-0.08, 0.03) |
| Executive and top management |  | ref | ref |  | ref | ref |
| **Household income per consumption unit** |  |  |  |  |  |  |
| 1st quintile (lowest) |  |  | **-0.10 (-0.18, -0.02)** |  |  | **0.30 (0.23, 0.37)** |
| 2nd quintile |  |  | 0.00 (-0.07, 0.07) |  |  | **0.08 (0.02, 0.14)** |
| 3rd quintile |  |  | -0.04 (-0.11, 0.02) |  |  | 0.05 (-0.01, 0.11) |
| 4th quintile |  |  | -0.01 (-0.06, 0.05) |  |  | -0.01 (-0.06, 0.04) |
| 5th quintile (highest) |  |  | ref |  |  | ref |
| **Paternal characteristics** |  |  |  |  |  |  |
| **Age** | 0.002  (-0.002, 0.006) | 0.001  (-0.003, 0.005) | 0.0007  (-0.003, 0.004) | **-0.008**  **(-0.01, -0.005)** | -0.007  (-0.01, -0.004) | -0.006  (-0.009, -0.003) |
| **Number of older siblings of the ELFE child in the household** |  |  |  |  |  |  |
| None | ref | ref | ref | ref | ref | ref |
| 1 sibling | **-0.08 (-0.13, -0.04)** | -0.08 (-0.13, -0.04) | -0.07 (-0.12, -0.03) | **0.20 (0.16, 0.24)** | 0.20 (0.16, 0.24) | 0.18 (0.14, 0.22) |
| ≥ 2 siblings | **-0.06 (-0.12, -0.01)** | -0.06 (-0.12, 0.00) | -0.04 (-0.10, 0.02) | **0.27 (0.22, 0.32)** | 0.27 (0.22, 0.32) | 0.21 (0.16, 0.26) |
| **Immigrant status** |  |  |  |  |  |  |
| Immigrant | **-0.15 (-0.28, -0.02)** | -0.13 (-0.27, 0.00) | -0.11 (-0.24, 0.03) | **0.28 (0.16, 0.40)** | 0.26 (0.14, 0.38) | 0.18 (0.06, 0.30) |
| Descendant of immigrants | -0.02 (-0.08, 0.03) | -0.02 (-0.07, 0.04) | -0.01 (-0.06, 0.05) | **0.15 (0.10, 0.20)** | 0.14 (0.09, 0.19) | 0.11 (0.06, 0.16) |
| Non-immigrant | ref | ref | ref | ref | ref | ref |
| **Education level** |  |  |  |  |  |  |
| Low | **-0.16 (-0.21, -0.11)** | -0.11 (-0.16, -0.05) | -0.10 (-0.16, -0.04) | **0.26 (0.21, 0.30)** | 0.20 (0.14, 0.25) | 0.17 (0.12, 0.22) |
| Medium | **-0.09 (-0.14, -0.03)** | -0.07 (-0.13, -0.01) | -0.07 (-0.13, -0.01) | **0.12 (0.07, 0.17)** | 0.10 (0.04, 0.15) | 0.09 (0.04, 0.15) |
| High | ref | ref | ref | ref | ref | ref |
| **Occupational category** |  |  |  |  |  |  |
| Manual worker, unemployed, student |  | **-0.16 (-0.24, -0.08)** | -0.14 (-0.22, -0.06) |  | **0.21 (0.14, 0.29)** | 0.15 (0.08, 0.22) |
| Clerck |  | **-0.06 (-0.12, 0.00)** | -0.05 (-0.11, 0.01) |  | **0.07 (0.01, 0.12)** | 0.03 (-0.03, 0.08) |
| Farmer and skilled blue-collar worker |  | -0.05 (-0.13, 0.03) | -0.03 (-0.11, 0.05) |  | **0.08 (0.01, 0.16)** | 0.02 (-0.05, 0.10) |
| Intermediate occupation |  | 0.04 (-0.02, 0.11) | 0.05 (-0.02, 0.11) |  | 0.01 (-0.05, 0.06) | -0.01 (-0.07, 0.05) |
| Executive and top management |  | ref | ref |  | ref | ref |
| **Household income per consumption unit** |  |  |  |  |  |  |
| 1st quintile (lowest) |  |  | **-0.12 (-0.20, -0.04)** |  |  | **0.34 (0.27, 0.42)** |
| 2nd quintile |  |  | -0.02 (-0.09, 0.05) |  |  | **0.11 (0.05, 0.17)** |
| 3rd quintile |  |  | -0.04 (-0.10, 0.03) |  |  | **0.08 (0.02, 0.14)** |
| 4th quintile |  |  | 0.005 (-0.05, 0.06) |  |  | -0.001 (-0.05, 0.05) |
| 5th quintile (highest) |  |  | ref |  |  | ref |

Model with maternal characteristics: Model 1 included maternal age, number of older siblings of the ELFE child in the household, single motherhood, immigrant status, education level; model 2 additionally included maternal occupational category; and model 3 additionally included household income. All models were adjusted for child sex and conurbation size as covariates and partner’s education level as a confounder.

Model with paternal characteristics: Model 1 included paternal age, number of older siblings of the ELFE child in the household, immigrant status, education level; model 2 additionally included paternal occupational category; and model 3 additionally included household income. All models were adjusted for child sex and conurbation size as covariates and partner’s education level and single motherhood as confounders.

Coefficients are interpreted when the variable appears the first time (in grey), but we showed all estimates for indication.

References

1.	Gleason PM, Boushey CJ, Harris JE, Zoellner J. Publishing nutrition research: a review of multivariate techniques--part 3: data reduction methods. Journal of the Academy of Nutrition and Dietetics. 2015;115(7):1072-82.

2.	Josse J, & Husson, F. missMDA: A Package for Handling Missing Values in Multivariate Data Analysis. . Journal of Statistical Software. 2016;70(1–31).

3.	Bournez M, Ksiazek E, Wagner S, Kersuzan C, Tichit C, Gojard S, et al. Factors associated with the introduction of complementary feeding in the French ELFE cohort study. Matern Child Nutr. 2018;14(2):e12536.
